# Overexpression of *OsDUF868.12* enhances salt tolerance in rice

**DOI:** 10.3389/fpls.2025.1458467

**Published:** 2025-01-29

**Authors:** Hao Chen, Jiale Wan, Jiali Zhu, Ziyi Wang, Caiyao Mao, Wanjing Xu, Juan Yang, Yijuan Kong, Xiaofei Zan, Rongjun Chen, Jianqing Zhu, Zhengjun Xu, Lihua Li

**Affiliations:** ^1^ State Key Laboratory of Crop Gene Exploration and Utilization in Southwest China, Rice Research Institute, Sichuan Agricultural University, Chengdu, China; ^2^ Crop Ecophysiology and Cultivation Key Laboratory of Sichuan Province, Sichuan Agricultural University, Chengdu, China

**Keywords:** *OsDUF868.12*, rice, abiotic stresses, oxidative stress, physiological and biochemical index

## Abstract

Excessive salt accumuln in soil is one of the most important abiotic stresses in agricultural environments. The Domain of Unknown Function 868 (DUF868) family, comprising 15 members in rice, has been identified in the protein family database. In this study, we cloned and functionally characterized *OsDUF868.12*, a member of the OsDUF868 family, to elucidate its role in rice response to salt stress. A series of experiments, including RT-qPCR, Agrobacterium-mediated transient transformation in tobacco for localization analysis, phenotypic characterization, physiological and biochemical index measurement, and leaf staining, were conducted to investigate the function of *OsDUF868.12* under salt stress. Transcriptional analysis revealed that *OsDUF868.12* exhibited the most significant response to low temperature and salt stress. Preliminary subcellular localization studies indicated that *OsDUF868.12* is localized in the cell membrane. Phenotypic Identification Experiments showed Overexpression lines of *OsDUF868.12* enhanced resistance to salt stress and increased survival rates, while knockout lines of *OsDUF868.12* were opposite. Physiological and biochemical assessments, along with leaf staining, demonstrated that overexpression of *OsDUF868.12* improved the activity against oxidative stress.under salt stress. Furthermore, overexpression of *OsDUF868.12* elevated the transcription levels of positively regulated salt stress-related genes. These findings suggest that overexpression of *OsDUF868.12* enhances rice tolerance to salt stress at the molecular level through a series of regulatory mechanisms. This study provides valuable insights into the functional roles of the DUF868 family in plant responses to abiotic stress.

## 1 Introduction

Rice is one of the main food crops in the world (Khan et al., 2021). According to the forecast of the Food and Agriculture Organization (FAO), the world population will reach 9 billion in 2050, which requires a further increase in global food production. In recent years, climate change has intensified, and extreme weather has occurred frequently, the impact of abiotic stresses on plants has become more serious (Fedoroff et al., 2010). Soil salinization will become one of the main factors affecting agricultural production in the coming decades.

Excessive salt accumulation in soil is one of the most important abiotic stresses in agricultural environments (Munns, 2002). Plants growing in saline-alkali soil will suffer from ion toxicity, oxidative damage and osmotic stress (Munns and Tester, 2008), which will significantly inhibit the vegetative growth, seed germination, yield and quality of rice (Quan et al., 2007). Improving the salt tolerance of rice can not only improve the yield and quality of rice, but also solve the problem of reducing the cultivated land area to a certain extent (Yang et al., 2023). Plants have evolved a variety of strategies to cope with salt stress, including ion homeostasis regulatory pathways, antioxidant system activation, osmoregulatory substance synthesis, etc. They form an interconnected signal network (Zhao et al., 2020), for example, there are a variety of CBL-interacting protein kinases (CIPKs) modules in plants, among which the most common pathway used to transmit salt stress signals is the SOS pathway (Salt Over Sensitive) (Zhu, 2002). The protein kinase SOS2 is mainly recruited and activated by calcium sensor SOS3 and then, SOS2 phosphorylates the Na^+^/H^+^ reverse transporter, SOS1, which is located on the cell membrane (Qiu et al., 2002), and finally reduces the absorption of Na^+^ by excreting it out of the cell. In addition, as a reverse transporter of Na^+^/H^+^ on the vacuole membrane, NHX1 can also transport cytoplasmic Na^+^ to the vacuole to prevent excessive concentration of Na^+^ in the cytoplasm and adverse effects it caused on cells (Barragán et al., 2012), and maintain cell cation homeostasis (Bassil et al., 2019). Finally reduces the absorption of Na^+^ by excreting it out of the cell.

DUF families are characterized by relatively conserved amino acid sequences and unknown functional domains (Finn et al., 2016). In the Pfam 35.0 database, the Pfam database contains 4795 DUF or UPF families, accounting for 24% of the total protein families in the Pfam database. DUF families are divided into different families based on their specific domains. Studies have shown that DUF families play an important role in plant growth, development, breeding and resistance to biotic and abiotic stresses (Lv et al., 2023). *OsRMC* mediates root development through jasmonic acid (JA) pathway, it also negatively regulates root curl and participates in stomatal development; in *Arabidopsis thaliana*, overexpression of *DUF761-1* could affect leaf development, shorten leaf length, root length, inflorescence and horn fruit, and reduce seed number; OsSAC1, containing two conserved DUFs—DUF4220 and DUF594, affects sugar accumulation in rice leaves (Serra et al., 2013; Zhu et al., 2018; Zhang et al., 2019). In addition, DUFs also contribute to plant abiotic stress response. Overexpression of *OsDSR2* could increase sensitivity to salt and simulated drought stress and reduce ABA sensitivity of rice. *OsSIDP301*, a member of the DUF1644 family, negatively regulates salt stress and grain size in rice, while *OsSIDP366*, another member of this family, positively regulates responses to drought and salt stresses in rice (Luo et al., 2014; Guo et al., 2016; Ge et al., 2022).

The DUF868 family is widely expressed in plants, but little research has been done on this family. In the study presented here, we showed that *OsDUF868.12*, which was named according to the order of distribution of DUF868 members on chromosomes in rice, could respond to a variety of abiotic stresses and overexpression of *OsDUF868.12* could enhance the salt tolerance of rice.

## 2 Material and methods

### 2.1 Plant materials and growth condition

Rice (*Oryza sativa* L. subsp. japonica cv. Nipponbare) seeds were used as wild type (WT) and materials for genetic transformation. In all experiments, the seeds were soaked with 2% (v/v) NaClO for 30 min, washed with sterile water and subsequently subjected to imbibition at 37°C for 3 days in the dark. Then, the germinated seeds were cultured in nutrient solution under a cycle of 16-h light at 28°C and 8-h dark at 25°C.

### 2.2 Construction of transgenic plants

The *OsDUF868.12* gene was amplified from WT and cloned into the pU1300 vector to obtain overexpression lines (*OsDUF868.12*-OE), and knockout lines (*OsDUF868.12*-KO) were obtained by using the clustered regularly interspaced short palindromic repeats (CRISPR-Cas9) technique. To obtain the expression profile of *OsDUF868.12*, the promoter sequence of 1500 bp of *OsDUF868.12* was obtained through the NCBI database and amplified from the rice genomic DNA, the pCAMBIA1305 vector was duplexed with Hind III and Noc I endonuclease, followed by recombination. To explore the subcellular localization of *OsDUF868.12*, its coding sequence was amplified from WT and, subsequently, recombination ligation was performed with the pCAMBIA2300-GFP vector, which was duplexed with Hind III and BamH I endonuclease. Finally, the transgenic rice plants were obtained by Agrobacterium-mediated genetic transformation, and the overexpression lines and knockout lines were identified by RT-qPCR and DNA amplification sequencing, respectively.

### 2.3 RNA extraction and quantitative real-time PCR analysis

To study the effect of abiotic stresses and ABA on the transcript accumulation of *OsDUF868.12*, three-leaf stage WT (Nipponbare) seedlings were used for stress treatments, including cold (4°C), hot (42°C), salt (150 mM NaCl), drought (20% w/v PEG6000) and ABA (50 μM). Leaves were taken at different periods (0 h, 0.5 h, 1 h, 2 h, 4 h, 8 h, 16 h, 24 h and 0 h treatment was the control group) to extract total RNA using Trizol reagent according to the manufacturer’s instructions. The reverse transcription was done using a PrimeScript™ RT reagent Kit with a gDNA Eraser kit, and the cDNA was stored at -20°C. The extracted cDNA was used as a template to analyze the transcript accumulation of *OsDUF868.12* under normal and abiotic stress conditions. The initial amount of template cDNA in each amplification reaction was 10 µg. At least three independent biological replicates were performed for each experiment and the rice Ubiquitin gene (Os01g0328400) was used for internal control for qPCR normalization. The 2^−ΔΔCT^ method was used to transform threshold cycle values (Ct) into normalized relative abundance values of mRNA. All primer pairs used were listed in **Supplementary Table S1**.

### 2.4 Histochemical GUS activity assay and subcellular localization of *OsDUF868.12*

Different tissues of the *OsDUF868.12*p: GUS transgenic plants were collected and detected following the previous method. The tissues were placed in a buffer containing 50 mM NaPO_4_ (pH 7.2), 5 mM K_3_Fe (CN)_6_, 5 mM K_4_Fe (CN)_6_, 0.1% (w/w) Triton-100, and 1 mm X-Gluc, and they were incubated overnight at 37°C and soaked tissues in 70% (v/v) ethanol for 5 min to stop the staining. Then, 95% (v/v) ethanol was added and removed chlorophyll completely. Finally, photos were taken with ZEISS stereo microscope.

The seeds of green fluorescent protein (GFP) transgenic plants were germinated, and the roots of 6-day-old seedlings were taken and placed on a slide, mashed, and stained with 10 µM FM4-64 cell membrane dye. After staining, the excess dye solution was cleaned, the cover glass was covered, and the expression of *OsDUF868.12*-GFP fusion protein was observed under the confocal laser microscope.

Individually cultured *Agrobacterium* containing the pCAMBIA2300-GFP vector and the membrane marker vector in liquid media until the logarithmic growth phase. Then, the *Agrobacteria* were centrifuged at 4000 rpm for five minutes. The collected *Agrobacteria* were resuspended in the resuspension solution to an OD_600_ of 0.5–1, mixed in a 1:1 ratio, and the mixture was injected into the lower epidermis of tobacco leaves after two hours. The results were observed under a laser confocal microscope 2 days later. The resuspension solution consisted of 100 ml containing 100 µL of 10 mM ACE, 1 ml of 0.2 M MgCl_2_, 5 ml of 0.2 M MES (pH 5.6), and 93.9 ml of ddH_2_O.

### 2.5 Transgenic lines treated with abiotic stress and exogenous plant hormone

In order to investigate the tolerance of transgenic lines to salt stress, the 2-day-old rice seedlings were cultured in a nutrient solution containing 150 mM NaCl for 6 days; and observed the growth inhibition status of shoot. Further, 2-week-old rice seedlings were cultured in a nutrient solution containing 200 mM NaCl for 6 days, followed by 10 days of recovery and calculated the survival rates. To investigate the resistance of transgenic rice to oxidative stress, the leaves of 2-week-old transgenic and WT seedlings were cut and placed in 2% (v/v) hydrogen peroxide (H_2_O_2_) solution for 2 days and observed the degree of leaf green fade. To investigate the response of transgenic lines to various exogenous plant hormones. The 2-day-old WT and transgenic lines were cultured in nutrient solution for 6 days, which contained 1 µM ABA, and observed the growth status.

### 2.6 Measurement of the content of chlorophyll

The content of chlorophyll was measured as previously described (Gao et al., 2020). The leaves were cut into small pieces and soaked in extract (acetone: ethanol = 2:1) for 2 days in the dark. Subsequently, the samples were centrifuged at 4000 rpm for 5 minutes and the supernatant was collected. The absorbance of the supernatant was measured at wavelengths of 663 nm and 645 nm, respectively. [Chla] = (12.7*A663−2.69*A645)*V/(1000*W). [Chlb] = (22.9*A645−4.68*A663) *V/(1000*W), [Chlt] = [Chla) + [Chlb]. V: the total volume of the extract, W: the weight of the sample.

### 2.7 Measurement of the physiological and biochemical indicators

In order to determine the activities of superoxide dismutase (SOD), peroxidase (POD), content of malondialdehyde (MDA) and content of proline (Pro), the 2-week-old seedlings were transferred to nutrient solution containing or without 200 mM NaCl for 2 days and the aerial parts of the plants were sampled. The 0.2 g sample of the aerial parts was ground into powder by liquid nitrogen, then 3 mL of 100 mM phosphate buffer (pH 7.8) was added and the sample was ground into a homogenate. The homogenate was centrifuged at 10,000×g for 10 min at 4°C and the supernatant was used for the assays.

In the SOD assay, 0.2 mL of supernatant was added to 4 mL 100 mM phosphoric acid solution (pH 7.8), 0.08 mL 1 mM EDTA-Na2, 0.27 mL 750 µM NBT, 0.27 mL 130 mM Met, and 0.27 mL 20 µM ribonucleotide in the experimental group. The supernatant was replaced with 0.2 mL PBS as control I and control II. Control I was placed in the dark, and the experimental group and control II were placed in light conditions for 15 min. Control I was used as a reference to adjust the zero and the absorbance at 560 nm was measured using an enzyme meter. In the POD assay, 0.2 mL of supernatant was added to 4 mL 100 mM phosphoric acid solution (pH 7.0), 2.3 µL of 0.2% (v/v) guaiacol, and 2 µL of 30% (v/v) hydrogen peroxide, and recorded the absorbance for 1 min at 470 nm. In the MDA content analysis, 0.1 mL of supernatant was added to 0.4 mL of 0.25% (w/v) Thiobarbituric acid (TBA), boiled for 15 min, and cooled in an ice bath for 5 min, the absorbance at both 532 and 600 nm was recorded for 1 min, respectively. In the content of Pro analysis, 10 mL of 3% sulfosalicylic acid, 10 mL acetic acid and 20 ml of 2.5% acid ninhydrin solution were mixed as reaction solution. 50 µL of supernatant was added to 1 ml of the reaction solution, and the absorbance at 520 nm was recorded.

In addition, Nitro blue tetrazolium (NBT) and 3’-diaminobenzidine (DAB) were used to detect the content of O_2_^-^ and H_2_O_2_ in the leaves, as previously described (Chen et al., 2021a). The isolated leaves were placed in DAB and NBT solutions overnight at 28°C under light and after staining, and soaked in 95% ethanol overnight to remove chlorophyll.

### 2.8 Bioinformatics and statistical analysis

The sequences of the *OsDUF868.12* gene were downloaded from The Rice Genome Annotation Project (RGAP) database. The cis-acting elements were found by using the PlantCARE to analyze the promoter sequence of *OsDUF868.12*. Members of DUF868 family in rice were found in the rice RGAP database, and then other members of the DUF868 family in *Zea mays*, *Arabidopsis thaliana* and *Solanum lycopersicum* were found in the ensemble plants database. These data were finally analyzed through a phylogenetic tree, all experiments were repeated three times. These data were processed and analyzed by the t-test, with *P* < 0.05 (*) and *P* < 0.01 (**) to be significantly different.

## 3 Results

### 3.1 Bioinformatics analyze of *OsDUF868.12*

**Figure 1:**
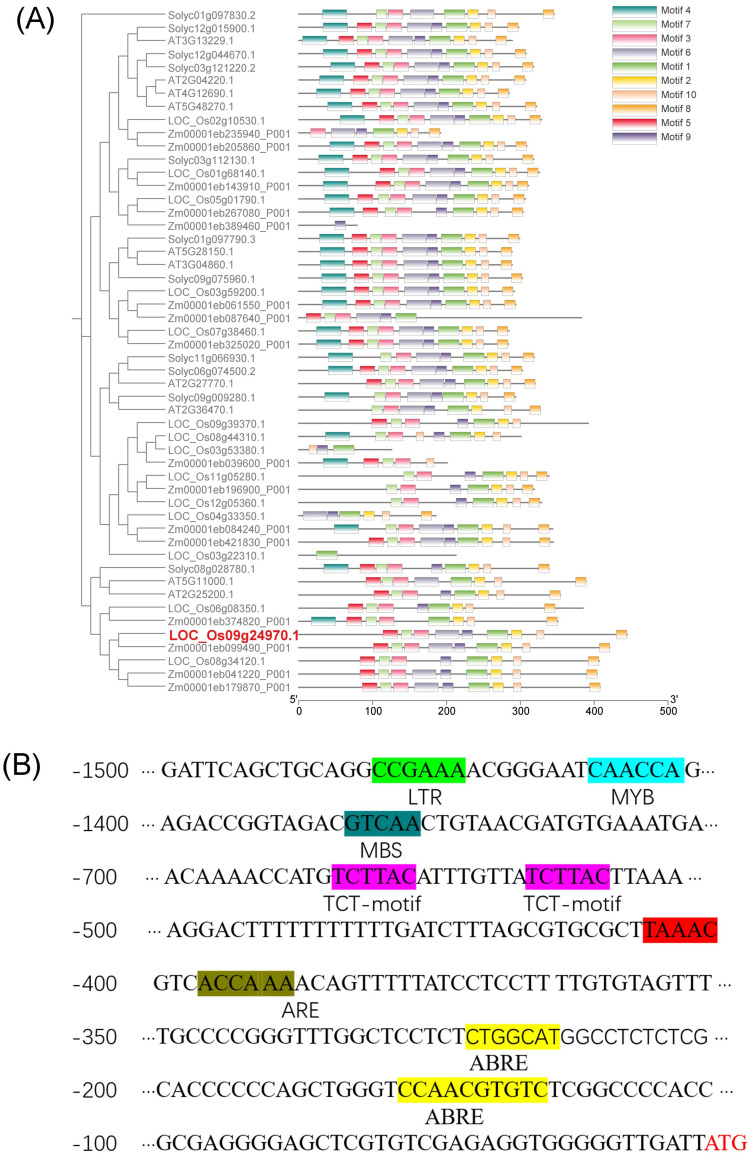
Bioinformatics analysis of *OsDUF868.12.* **(A)** Analysis of evolutionary relationship of DUF868 family. **(B)** Analysis of promoter sequence of *OsDUF868.12*.

The genetic information of *OsDUF868.12* was obtained through the RGAP database (rice.uga.edu/cgi-bin/ORF_infopage.cgi), and the results showed that *OsDUF868.12* is located on chromosome 9 with an open reading frame of 1335 bp and no introns, encoding 444 amino acid residues. In order to understand the phylogenetic relationship of *OsDUF868.12*, other DUF868 family members from *Oryza sativa*, *Zea mays*, *Arabidopsis thaliana* and *Solanum lycopersicum* were selected for phylogenetic analysis in this study. The results showed that *OsDUF868.12* (LOC_Os09g24970.1) had the closest evolutionary relationship with Zm00001eb099490_P001. Meanwhile, the motifs of protein sequences of DUF868 members were analyzed by using MEME software, and a total of 10 motifs were identified. The sequence of *OsDUF868.12* had 9 motifs, and these motifs existed in the majority of DUF868 members, indicating that *OsDUF868.12* was evolutionarily conservative (**Figure 1A**). Meanwhile, through the PlantCARE promoter online analysis tool, the 1500 bp promoter elements upstream of the ATG sequence (start codon sequence) of *OsDUF868.1*2 was analyzed. It was found that in addition to a large number of basic promoter elements such as CAAT-box, TATA-box, there were several cis-acting elements related to abiotic stresses (**Figure 1B**), such as TCT-motif (light-responsive element), ABRE-motif (abscisic acid-responsive cis-element), LTR-motif (low-temperature-responsive element), MBS-motif (drought-induced MYB-binding element), ARE-motif (anaerobic-inducible element).

### 3.2 *OsDUF868.12* could respond to abiotic stress

**Figure 2:**
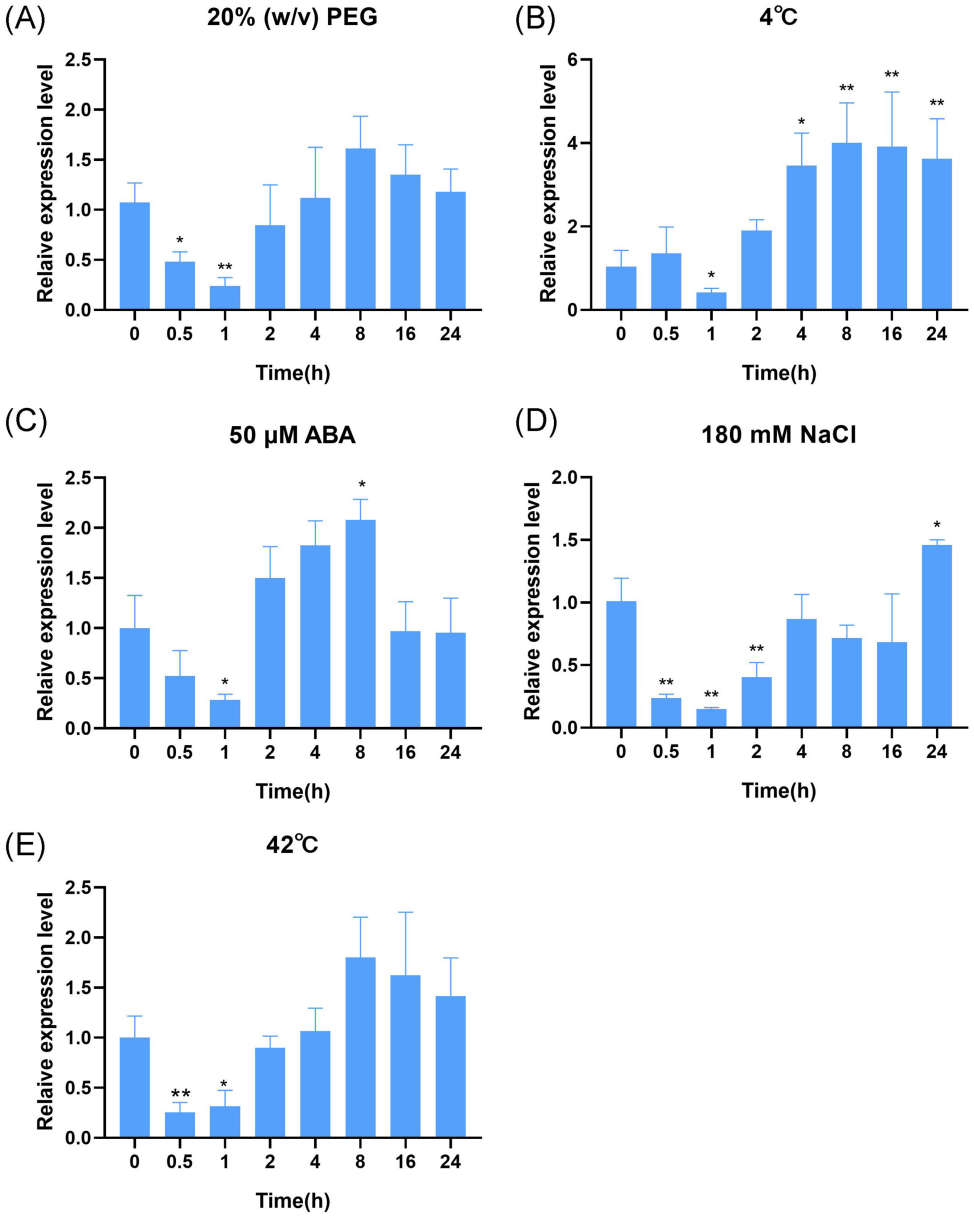
The transcript levels of *OsDUF868.12* in WT at the three-leaf stage under abiotic stress treatments. **(A)** 20% (W/V) PEG6000; **(B)** 4°C; **(C)** 50 µM ABA; **(D)** 180 mM NaCl; **(E)** 42°C. Data show the mean ± SD of three replicates. Asterisks indicate significant differences between 0 h and the rest of the time using t-test (*P<0.05, **P<0.01).

To understand the response of *OsDUF868.12* to different types of abiotic stress. WT were treated with various abiotic and ABA at the three-leaf stage for 24 h and sampled, which were analyzed by RT-qPCR and the control group at the corresponding time was used as reference. We found that the transcript level of *OsDUF868.12* fluctuated from a minimum of 0.24-fold under 20% (w/v) PEG treatment (**Figure 2A**) to a maximum of 4.00-fold under 4°C treatment (**Figure 2B**). In addition, we found that its transcript level was raised to 2.08, 1.46 and 1.80-fold under 50 µM ABA, 180 mM NaCl and 42°C (**Figures 2C–E**) treatment, respectively. Based on the significance analysis, we preliminarily inferred that *OsDUF868.12* is most likely to respond to cold and salt stress.

### 3.3 Histochemical *GUS* staining *and* subcellular localization of *OsDUF868.12*

**Figure 3:**
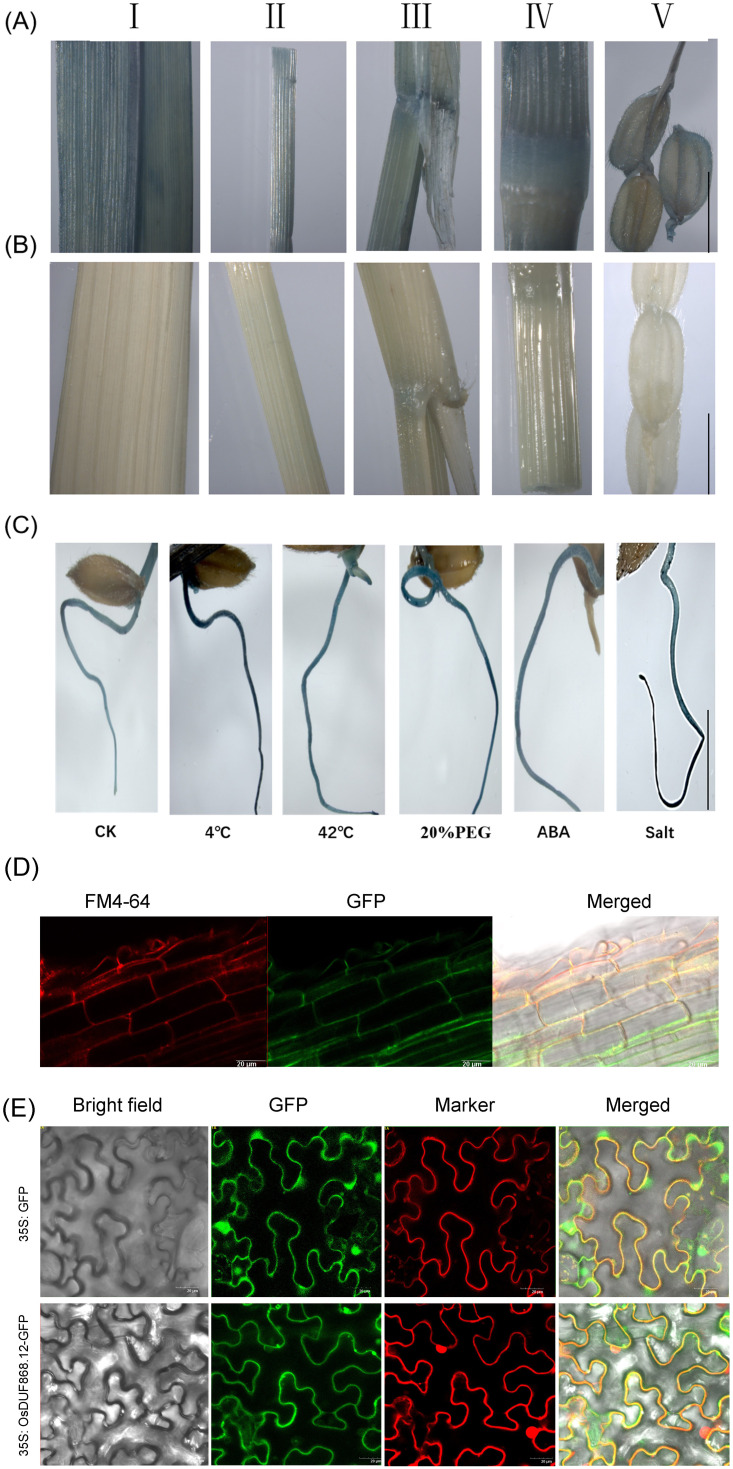
Histochemical GUS activity and subcellular localization. **(A)** GUS staining of different tissues of *OsDUF868.12*pro: GUS plants. (I: leaf; II: stem; III: leaf ligule; IV: internode; V: young spikelet hull). bar, 1 cm. **(B)** GUS staining of different tissues of WT (as a negative control for Figure. **A**). bar, 1 cm. **(C)** GUS staining of 5-day-old root of *OsDUF868.12*pro: GUS plants under abiotic stresses. bar, 2 cm. **(D)** Subcellular localization of *OsDUF868.12* in root of rice. bar, 20 μm. **(E)** Subcellular localization of *OsDUF868.12* in tobacco cells. bar, 20 μm.

In order to analyze the expression pattern of *OsDUF868.12 in situ*, *OsDUF868.12*pro: GUS transgenic plants were constructed. Transgenic lines and WT (**Figures 3A, B**) were sampled and used for detection of β-glucuronidase (GUS) activity. The staining of different tissue sites, such as stem, leaf, ligule, internode, spike, and root, indicated that *OsDUF868.12* could widely expressed in different tissues. In addition, in order to understand the role of this gene in stress response, 5-day-old transgenic seedlings were treated with 4°C, 42°C, 180 mM NaCl, 20% (w/v PEG) and 50µM ABA for 12 h and stained, it showed that the root staining was deepened in different degrees. Among them, the root staining was deepest under 4°C and salt treatments (**Figure 3C**), further implying that the gene could respond to a variety of abiotic stresses.

In order to explore the subcellular localization of *OsDUF868.12*, GFP transgenic plants were constructed and the roots of plant seedlings were stained by FM4-64 staining solution, which was used as cell membrane dye, and *OsDUF868.12* was localized in the cell membrane (**Figure 3D**). At the same time, we conducted subcellular localization experiments using *Agrobacterium*-mediated transient transformation in tobacco. The result also showed that *OsDUF868.12* was localized in the cell membrane (**Figure 3E**), which was consistent with its localization in the root.

### 3.4 Overexpression lines of *OsDUF868.12* improve, but knockout lines reduce salt stress tolerance of rice

**Figure 4:**
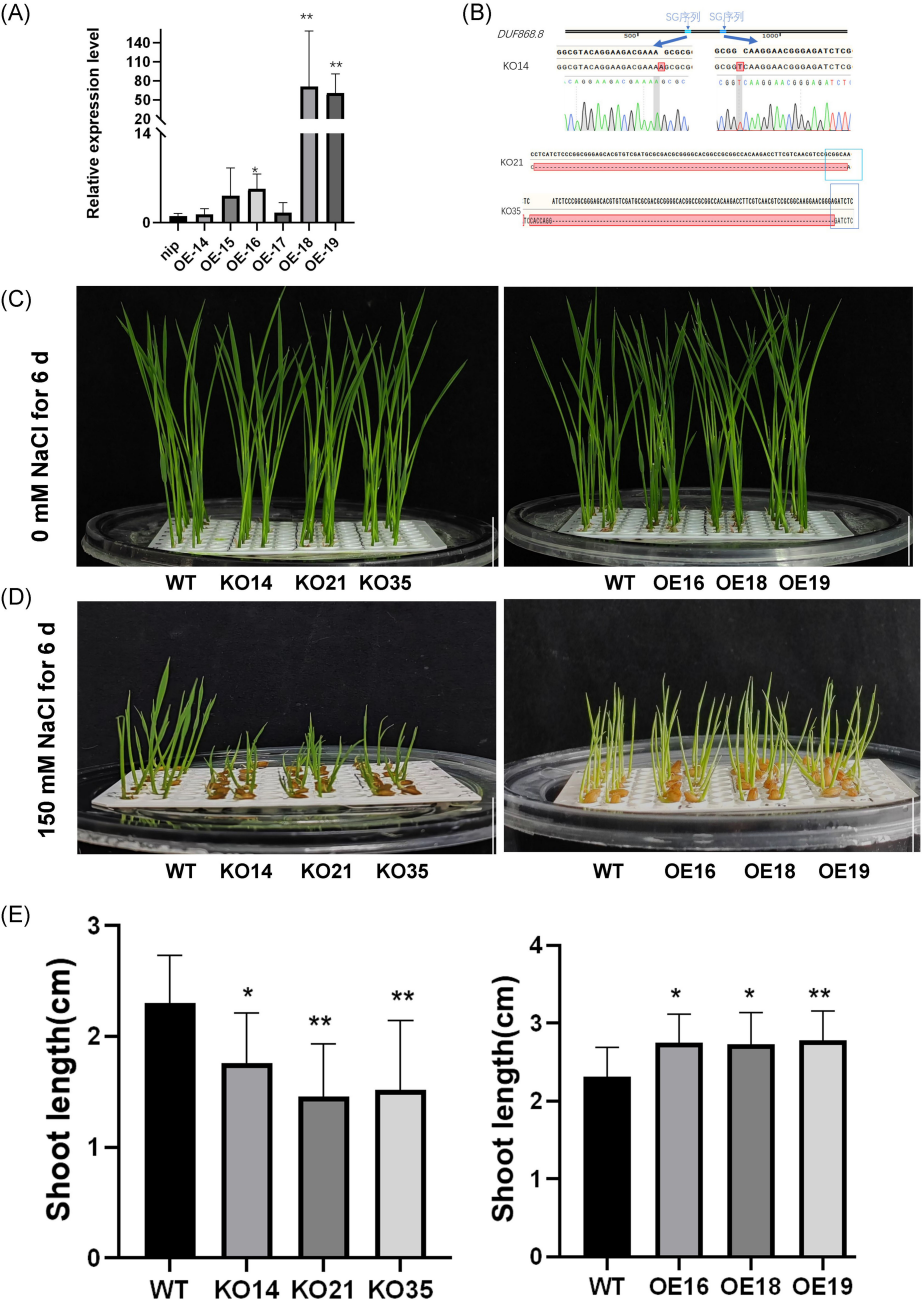
Detection of transgenic lines and growth inhibition experiments under 150 mM NaCl treatment. **(A)** The transcript levels of the overexpression lines were detected by RT-qPCR. **(B)** Detection of knockout sites. **(C)** Plant height of 2-day-old transgenic lines and WT after 6 days of growth under normal nutrient solution. bar, 1cm. **(D)** Plant height of 2-day-old seedlings after 6 days of growth under 150 mM NaCl. bar, 1 cm. **(E)** Statistics of plant height of transgenic lines and WT. Data show the mean ± SD of three replicates. Asterisks indicate significant differences between transgenic lines and WT using t-test (*P<0.05, **P<0.01).

In order to investigate the role of *OsDUF868.12* in the regulation of salt stress tolerance, overexpression and knockout lines were constructed, and the transgenic plants were identified by RT-qPCR and PCR. Three independent overexpression lines (OE16, OE18, and OE19) and three independent knockout lines (KO14, KO21, and KO35) were selected for subsequent experiments (**Figures 4A, B**). In the growth inhibition experiment, the 2-day-old transgenic and WT seedlings were grown in the nutrient solution containing or without 150 mM NaCl for 6 days. There was no significant difference between the transgenic and WT seedlings in the control group (**Figure 4C**). We found that under 150 mM NaCl treatment, the shoot length of knockout lines was significantly lower than WT under salt stress, but the overexpression lines had a better growth status than WT (**Figures 4D, E**).

**Figure 5:**
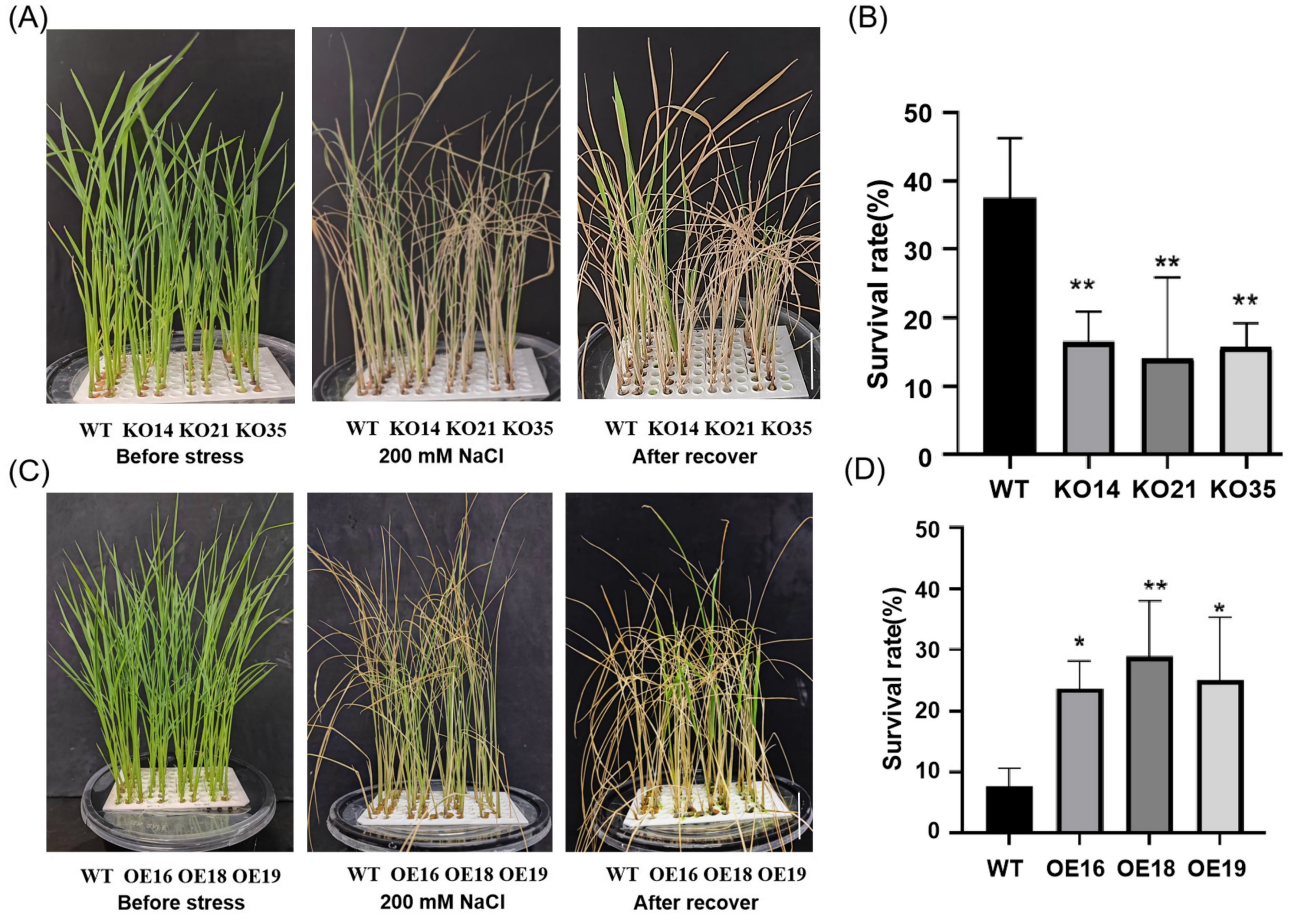
Survival experiment of transgenic lines and WT under 200 mM NaCl treatment. **(A)** Phenotype of 2-week-old WT and knockout lines before and after 200 mM NaCl treatment for 6 days and recovery for 15 days. bar, 5 cm. **(B)** Survival rate of WT and knockout lines after 200 mM NaCl treatment. **(C)** Phenotype of 2-week-old WT and the overexpression lines before and after 200 mM NaCl treatment for 6 days and recovery for 15 days. bar, 5 cm. **(D)** Survival rate of WT and knockout lines after 200 mM NaCl treatment. Data show the mean ± SD of three replicates. Asterisks indicate significant differences between transgenic lines and WT using t-test (*P<0.05, **P<0.01).

Furthermore, 2-week-old transgenic and WT seedlings were treated with 200 mM NaCl and the survival rates were observed. We found that after 6 days of treatment, compared with WT, the knockout lines exhibited more severe salt damage phenomena, manifested by more severe leaf chlorosis (**Figure 5A**), while the overexpression lines were opposite (**Figure 5C**). After recovery for 15 days, the survival rate of the knockout lines was significantly lower than WT (**Figure 5B**), but that of the overexpression lines were higher (**Figure 5D**). In summary, the knockout of *OsDUF868.12* significantly reduced the tolerance of rice seedlings to salt stress, and overexpression of *OsDUF868.12* significantly increased the tolerance of rice seedlings to salt stress.

### 3.5 Overexpression of *OsDUF868.12* decreases the sensitivity of rice to exogenous ABA

**Figure 6:**
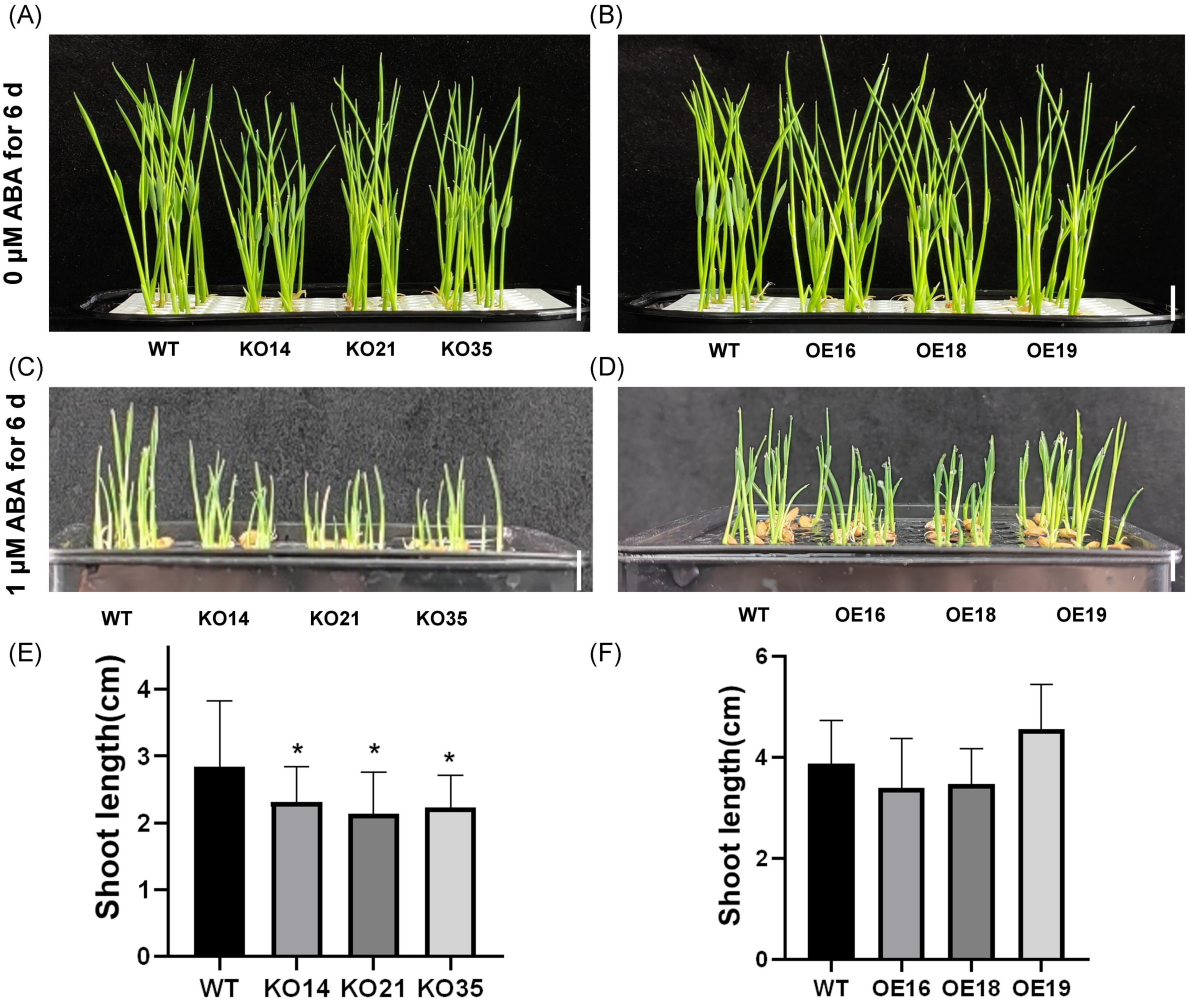
Inhibition of plant height of 2-day-old seedlings by ABA. **(A, B)** Plant height of 2-day-old seedlings after 6 days of growth under normal nutrient solution. bar, 1 cm. **(C, D)** Plant height of 2-day-old seedlings after 6 days of growth under 1 µM ABA. bar, 1cm. **(E, F)** Statistics of plant height for Figures **(C, D)**. Data show the mean ± SD of three replicates. Asterisks indicate significant differences between clines and WT using t-test (*P<0.05).

ABA is an important signaling molecule in plants in response to adversity stress, which could reduce salt stress damage by reducing Na^+^/K^+^ in plants, inducing the accumulation of osmotic substances and alleviating damage caused by osmotic and various ionic stresses (Qiao et al., 2023). To investigate whether *OsDUF868.12* regulates salt stress tolerance of rice in ABA-dependent pathways. We first explored the response of *OsDUF868.12* transgenic plants to exogenous ABA during the germination period. The 2-day-old transgenic and WT seedlings were grown in nutrient solution containing or without 1 µM ABA for 6 days. There was no significant difference between transgenic and WT seedlings in the control group (**Figures 6A, B**). Under exogenous ABA treatment, the shoot length of the knockout lines was significantly lower than WT, while there was no significant difference between the overexpression lines and WT (**Figures 6C–F**). The results showed that overexpression of *OsDUF868.12* could decrease the sensitivity of rice to exogenous ABA compared to the knockout of *OsDUF868.12*.

### 3.6 Overexpression of *OsDUF868.12* decreases the degree of cell damage under salt stress

**Figure 7:**
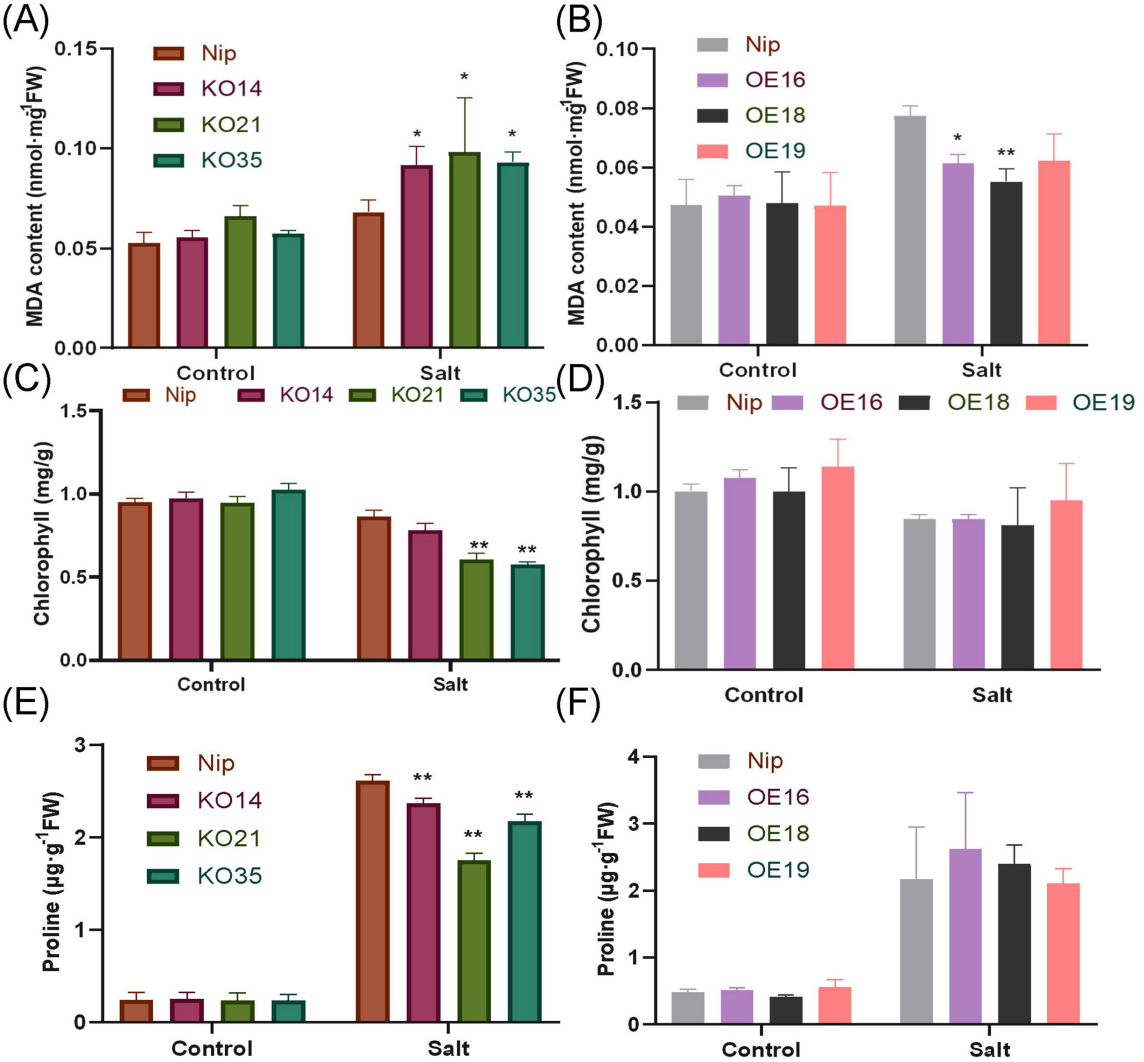
Analysis of MDA, chlorophyll and Proline content of WT and transgenic plants under normal and 200 mM NaCl-treated conditions. **(A, B)** MDA content, **(C, D)** chlorophyll content, **(E, F)** Pro content. Data show the mean ± SD of three replicates. Asterisks indicate significant differences between transgenic lines and WT using t-test (*P<0.05,**P<0.01).

Measurement of MDA and chlorophyll content could be used to assess the degree of damage degree of plant cells. In this study, there was no difference in the MDA content between transgenic lines and WT under normal condition. After 200 mM NaCl stress treatment for 3 days, the MDA content of the knockout lines was significantly higher than WT, and the overexpression lines were opposite (**Figures 7A, B**). Similar results were also reflected in experiments for the determination of chlorophyll content. After salt stress treatment, compared with WT, less chlorophyll was retained in the knockout lines and there were significant differences in KO21 and KO35, but there was no significant difference between the overexpressed lines and WT (**Figures 7C, D**). Proline is an important osmoregulatory substance, which is commonly used to protect cells. In this study, no significant difference in proline content in control group. After treatment, the accumulation of proline in the knockout lines was significantly lower than that of WT (**Figures 7E, F**). Above results illustrated that overexpression of *OsDUF868.12* could reduce the cell damage caused by salt stress.

### 3.7 *OsDUF868.12* enhances tolerance of rice to oxidative stress

**Figure 8:**
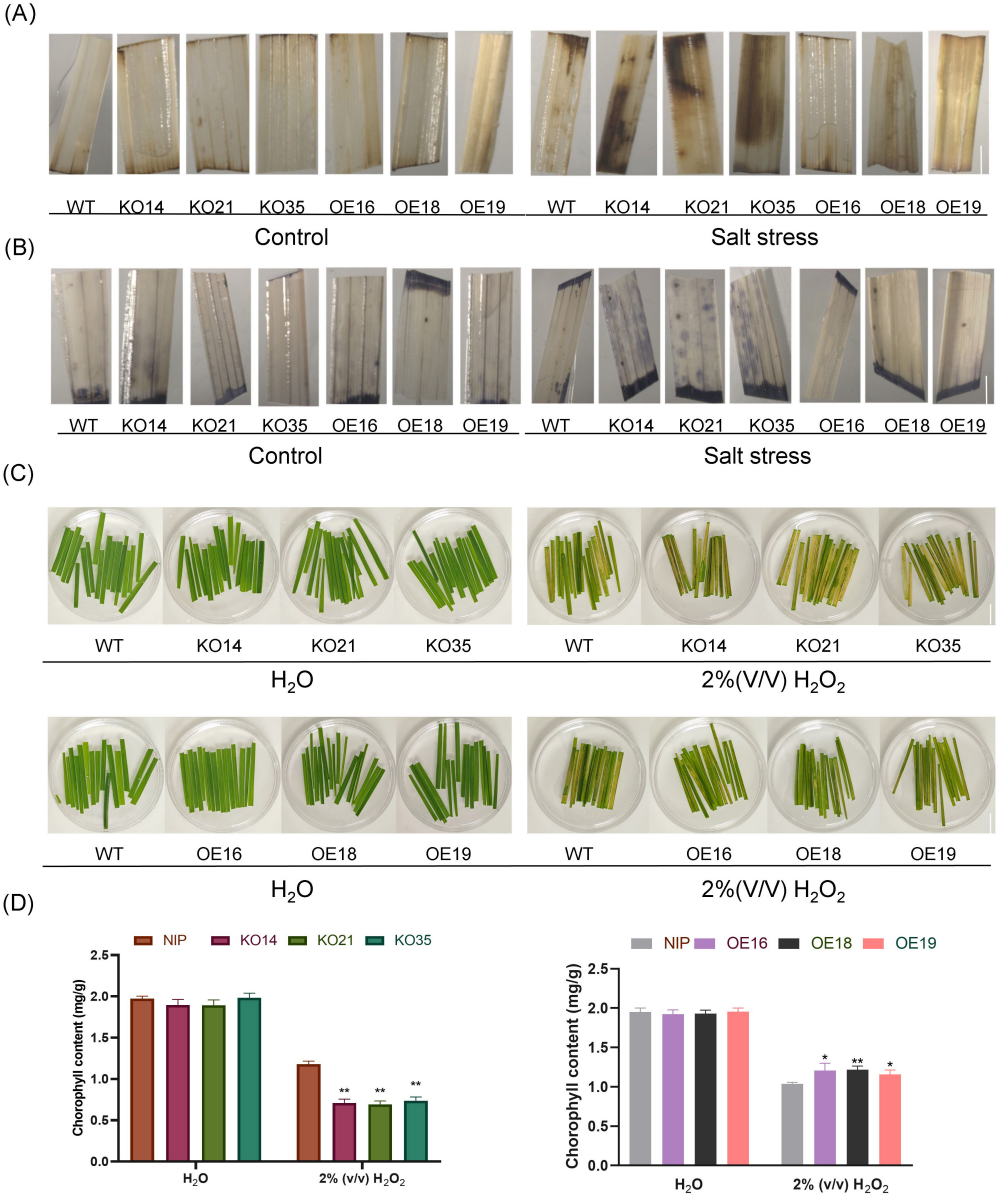
The accumulation of ROS level in leaves of 2-week-old transgenic plants of *OsDUF868.12* and WT before and after 2 days of treatment using 200 mM NaCl and oxidation experiment of H_2_O_2_. **(A)** DAB staining. bar, 3 mm. **(B)** NBT staining. bar, 3mm **(C)** 2% (v/v) H_2_O_2_ treated detach leaves for 2 days. bar, 1 cm. **(D)** The chlorophyll content of leaves before and after 2 days H_2_O_2_ treatment. Data show the mean ± SD of three replicates. Asterisks indicate significant differences between transgenic lines and WT using t-test (*P<0.05, **P<0.01).

ROS, such as hydrogen peroxide (H_2_O_2_) and superoxide anion radicals (O_2_^-^), are produced and accumulated in plants under abiotic stress, and excessive accumulation of ROS is harmful to the plant. In this study, NBT and DAB staining were used to assess the accumulation of ROS in 2-week-old seedlings before and after 200 mM NaCl stress treatment. We found there was no significant difference in the control group. After 2 days of 200 mM NaCl treatment, compared with WT, more blue-purple spots and more severe surface browning appeared on the leaves of the knockout lines (**Figures 8A, B**), indicating that the knockout lines accumulated more ROS than WT, while the overexpression lines were opposite. Meanwhile, we treated 0.1g of leaves from WT and transgenic lines by using H_2_O_2_. After being treated with 2% (v/v) H_2_O_2_ for 2 days, the degree of leaf chlorosis of the overexpression lines was lower than that of WT, while the knockout lines were the opposite (**Figure 8C**). We further extracted chlorophyll from the leaves using extract (acetone: ethanol = 2:1). There was no significant difference between transgenic and WT seedlings in the control group. In the treated group, the chlorophyll content of the knockout lines was significantly lower than that of WT, while the chlorophyll content of the overexpression lines was significantly higher than that of WT (**Figure 8D**), indicating that the overexpression lines were more resistant to H_2_O_2_. Above results indicated that overexpression of *OsDUF868.12* could increase the oxidative stress tolerance of rice.

### 3.8 Overexpression of *OsDUF868.12* enhances ROS scavenging enzyme activity

**Figure 9:**
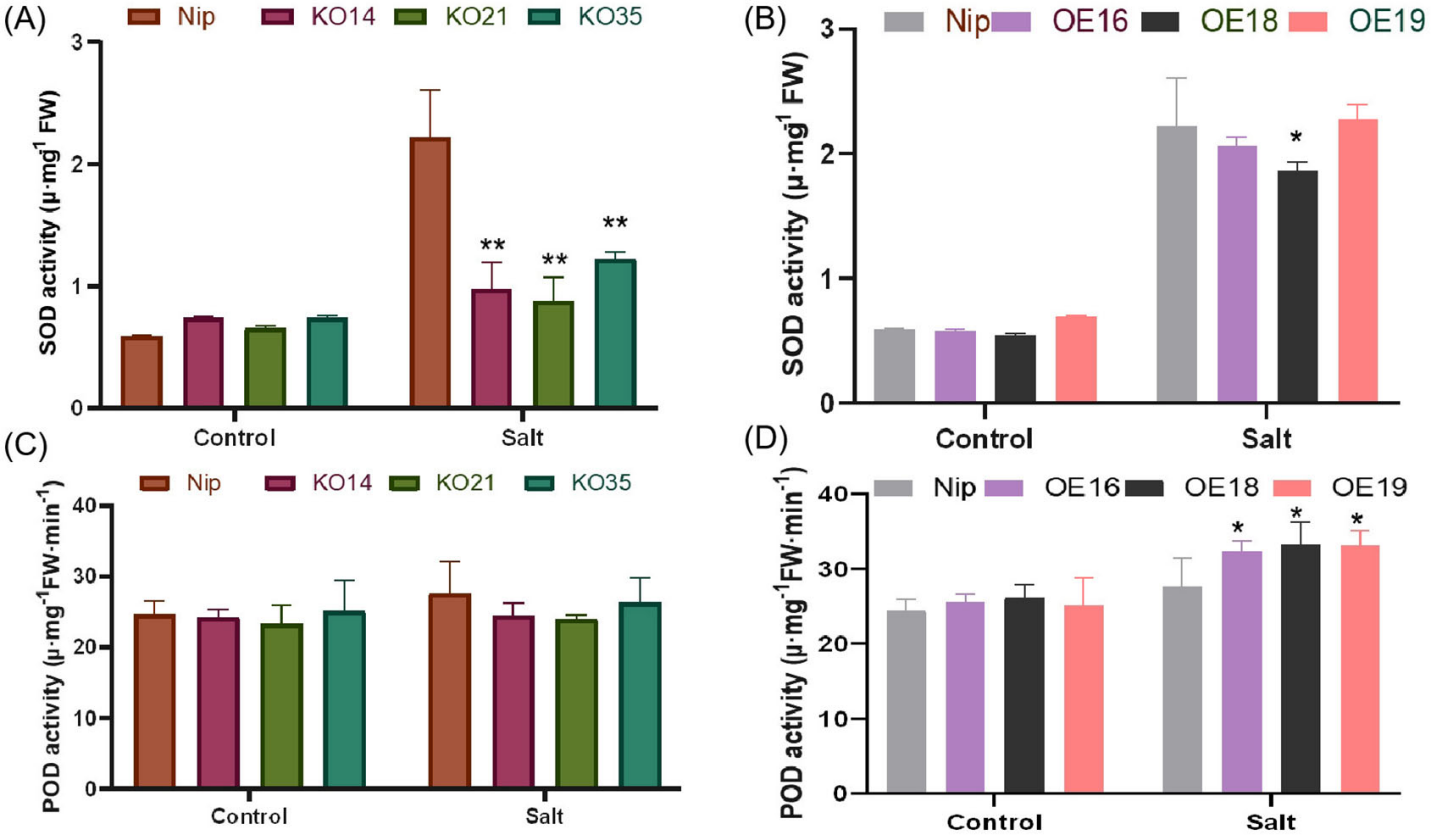
Analysis of SOD and POD activity. **(A, B)** SOD activity. **(C, D)** POD activity. Data show the mean ± SD of three replicates. Asterisks indicate significant differences between transgenic lines and WT using t-test (*P<0.05, **P<0.01).

In order to explore whether the differences in ROS accumulation between WT and transgenic lines were related to ROS-scavenging enzyme activities, the activities of SOD and POD were assayed, which are indicators were used to assess superoxide dismutase and peroxidase activities, respectively. Under normal condition, there was no significant difference in SOD and POD activities between WT and transgenic seedlings. After 3 days of salt stress treatment, both SOD and POD activities increased, but SOD activity of the knockout lines was significantly lower than WT (**Figure 9A**), while no significant difference was found between the overexpression lines and WT (**Figure 9B**). The POD activity of the overexpression lines was significantly higher than WT (**Figure 9C**), while that of the knockout lines did not change much (**Figure 9D**). In the previous study, it was also found that the accumulation of ROS in the overexpression lines was lower than that in WT and the knockout lines. Above results indicated that overexpression of *OsDUF868.12* could be able to increase the activity of the ROS-scavenging system, thereby reducing the accumulation of ROS and ultimately enhance salt stress tolerance.

### 3.9 Regulation of transcript accumulation of stress-related genes in transgenic lines of *OsDUF868.12*

**Figure 10:**
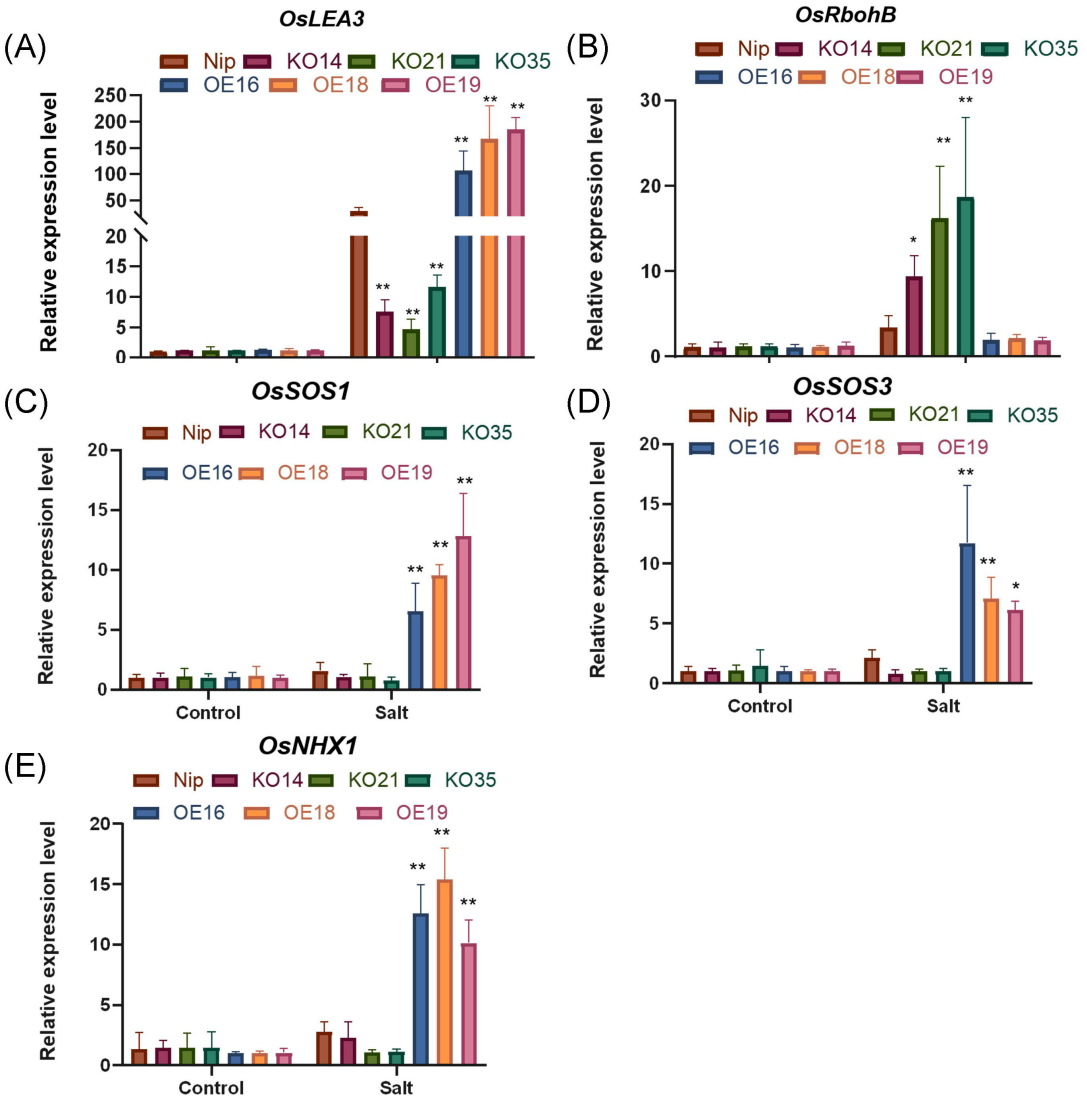
Transcript levels of stress-related genes in transgenic lines and WT treated with 200 mM NaCl for 8 (h) **(A)** *OsLEA3*. **(B)** *OsRbohB*. **(C)** *OsSOS3*. **(D)** *OsSOS1*. **(E)** *OsNHX1.* Data show the mean ± SD of three replicates. Asterisks indicate significant differences between transgenic lines and WT using t-test (*P<0.05, **P<0.01).

To further investigate the possible molecular mechanisms of salt tolerance in *OsDUF868.12*, we determined the transcript accumulation of some stress-responsive genes, such as *OsLEA3*, encoding a late embryogenesis abundant protein, which is a protective protein with a small molecular weight (Moons et al., 1997); OsRbohb, a respiratory burst oxidase homologue, has ROS-generating activity and its expression is mediated by stresses such as salt and drought (Wang et al., 2016); OsSOS1 and OsSOS3 are two critical proteins in the SOS pathway, responsible for Na^+^ extrusion at the plasma membrane and sensing cytosolic calcium signals, respectively (Gupta et al., 2021); while *OsNHX1* encodes a Na^+^/H^+^ antiporter located on the vacuolar membrane (Fukuda et al., 2011). After treatment with 200 mM NaCl for 8 h, the transcript levels of *OsLEA3* gene were significantly higher in the overexpression lines and lower in the knockout lines than WT (**Figure 10A**). The transcript levels of *OsRbohB* were significantly higher in the knockout lines and slightly lower in the overexpression lines than WT (**Figure 10B**). In addition, the transcript levels of *OsSOS1*, *OsSOS3*, and *OsNHX1* were significantly higher than WT in overexpression lines and lower than WT in the knockout lines (**Figures 10C–E**).

## 4 Discussion

Soil salinization is one of the important elements limiting the development of agriculture, and the effects of salt stress on plants mainly include osmotic stress, specific ionic toxicity, nutrient imbalance and ROS (Abbasi et al., 2016). In previous studies, many genes were found to be involved in plant response to salt stress, genes such as *GmCBSDUF3*, *AtRH17*, *OsDUF6* and *BrOAT1* can increase plant tolerance to salt stress through positive regulation (Jung et al., 2013; Seok et al., 2020; Chen et al., 2021b; Lv et al., 2023); *MdERF4*, *GhVIM28*, and *CYSTM3* can also decrease plant tolerance to salt stress through negative regulation (An et al., 2018; Xu et al., 2019; Yang et al., 2024).

Members of DUF families of proteins have been reported to play important roles at various stages of plant growth. In Arabidopsis thaliana, DUF246 could affect male fertility and the biosynthesis of pectic arabinogalactans. In Populus tremula (Stonebloom et al., 2016), DUF266 could increase cellulose content, reduce recalcitrance (Yang et al., 2017), and enhance biomass production. In rice, DUF1618 could induce hybrid incompatibility (Shen et al., 2017).

In this study, we identified *OsDUF868.12* of the OsDUF868 protein family member, which encodes a protein localized to the cell membrane. The results of GUS staining and changes in transcript levels of *OsDUF868.12* in WT under abiotic stresses indicate that *OsDUF868.12* mainly responds to cold and salt stress.

To further understand the role of *OsDUF868.12* in abiotic stress responses, we constructed overexpression and knockout lines. Under the culture of nutrient solution containing 150 mM NaCl, the growth state of the overexpression lines was less inhibited than WT, showing higher plant height, but the growth state of the knockout lines was more inhibited than WT. Meanwhile, under the culture of nutrient solution containing 200 mM NaCl, we found that the overexpression lines exhibited slower leaf chlorosis and the survival rates were higher than WT, while the knockout lines were opposite. Above results preliminarily indicated that *OsDUF868.12* could be able to increase the tolerance of rice to salt stress.

ROS has been identified as a class II small molecule that mediates the response to various abiotic stresses such as flooding, heavy metals, and high salinity. Under normal conditions, the generation of ROS at low levels can serve as intercellular signaling molecules, inducing responses in the antioxidant defense system. Conversely, the accumulation of excess ROS can lead to severe oxidative damage, impairing normal biological functions (Hasanuzzaman et al., 2020). It is produced by molecular oxygen and can be scavenged by superoxide dismutase, peroxidase, catalase and glutathione reductase (GR), which are essential for ROS homeostasis. NBT and DAB are used to determine the levels of O_2_^-^ and H_2_O_2_, respectively (Steffens et al., 2013). We found that under salt stress treatment, the overexpression lines had both lighter NBT and DAB staining compared to WT, while the knockout lines were opposite, demonstrating that the overexpression lines showed less accumulation of ROS under salt stress, while the knockout lines of *OsDUF868.12* showed deeper accumulation of ROS. MDA is used as an indicator of the degree of ROS-induced cell membrane damage and lipid peroxidation, for example, transgenic tobacco lines of *EsSPDS1* showed less accumulation of malondialdehyde in parallel with less ROS accumulation compared to WT (Zhou et al., 2015). In this study, the overexpression lines were found to have lower MDA assay under salt stress condition, which was consistent with the trend of less ROS accumulation. Changes in chlorophyll content are usually the opposite of changes in ROS. Under drought stress, overexpression lines of *CYP71D8L* had less ROS and more chlorophyll compared to WT (Zhou et al., 2020). In this experiment, compared with WT, the chlorophyll content of the knockout lines decreased more under salt stress. In addition, Proline is an important osmoregulatory substance, and the accumulation of it could improve the tolerance of plants to osmotic stress (Kishor et al., 1995), which further improves the tolerance of plants to salt stress. In this study, the overexpression lines accumulated more content of proline compared to WT. These results demonstrated that *OsDUF868.12* was able to improve antioxidant and salt stress tolerance in rice.

Under biotic and abiotic stresses, intracellular ROS homeostasis is disrupted by either enhanced ROS generation capacity or diminished ROS scavenging capacity (Wan et al., 2023), so increasing antioxidant enzyme activities to reduce ROS overproduction is the most effective way to improve plant resistance to abiotic stresses (Panda et al., 2021). Overexpression of *ThbHLH1* and overexpression of *zmlbd5* increased POD and SOD activities, thereby reducing the accumulation of ROS (Ji et al., 2016; Xiong et al., 2022). In this experiment, we found that the enzyme activities of transgenic lines of *OsDUF868.12* were not significantly different from those of WT under normal culture conditions, whereas after salt stress treatment, the POD activity was slightly lower than that of WT in the knockout lines, whereas the enzyme activity was significantly higher than WT in the overexpression lines. The SOD activity was significantly lower than WT in the knockout lines, whereas there was no significant difference between the overexpression lines and WT. These results demonstrated that *OsDUF868.12* could be able to improve the salt tolerance of rice by increasing the activities of SOD and POD.

ABA, the important stress hormone, is known to regulate different events of plant development in response to adverse environmental conditions and enable plants to withstand abiotic stresses. In general, increased tolerance to abiotic stresses and increased sensitivity to ABA in transgenic plants are correlated. In wheat, the overexpression of *TaASR1-D* enhanced tolerance to several abiotic stresses while enhancing sensitivity to ABA (Qiu et al., 2021). In this experiment, overexpression of *OsDUF868.12* increased salt tolerance and decreased ABA sensitivity, which is consistent with the fact that the overexpression of *OsPP108* increased salt tolerance and decreased ABA sensitivity, in Arabidopsis thaliana (Singh et al., 2015). Therefore, it is initially hypothesized that *OsDUF868.12* negatively regulates ABA signaling like *OsPP108*.

LEA is a class of protective proteins with small molecular weight, and a large number of studies have shown that it not only enhances osmoregulation, but also protects the function of proteins and enzymes thereby positively regulating plant resistance to abiotic stresses (Boucher et al., 2010; Liu et al., 2010; Su et al., 2011; Furuki and Sakurai, 2014). R proteins are a class of respiratory burst oxidase homologs, the large production of this enzyme leads to elevated ROS in plants (Foreman et al., 2003). The transcript level of *OsLEA3* gene in the overexpression lines was higher than WT after salt treatment, which was consistent with overexpression being more resistant to salt stress. The transcript level of *OsRbohB* in the overexpression lines was lower than WT, similarly consistent with the fact that the overexpression lines accumulated less ROS after salt stress treatment compared to WT.

OsSOS1 and OsSOS3 are responsible for Na^+^ efflux from the plasma membrane (Qiu et al., 2002), OsNHX1 promotes Na^+^ to enter the vesicle membrane, which plays an important role in ion homeostasis (Wang et al., 2022). They were significantly differentially expressed between overexpression plants and the knockout lines after salt stress treatment. The transcript levels of these genes in the overexpression lines were significantly higher than WT after stress treatment, while their transcription levels were slightly lower in the knockout lines. The tolerance to salt stress in the overexpression lines of O*sDUF868.12* was inextricably linked to the up-regulation of these salt tolerance genes.

## 5 Conclusion

This study focused on the biological function of *OsDUF868.12* under salt stress treatment. The results showed that the overexpression lines were more tolerant to salt stress, while the knockout lines were more sensitive to salt stress, suggesting that overexpression of *OsDUF868.12* could improve salt stress tolerance of rice. These findings provide a reference for further in-depth study of the *OsDUF868.12* and the development of other members of the DUF868 protein family.

## Data Availability

The original contributions presented in the study are included in the article/**Supplementary Material**. Further inquiries can be directed to the corresponding author.
