# Supplementary material for: Overexpression of OsDUF868.12 enhances salt tolerance in rice
Source: Front Plant Sci. 2025 Jan 29;16:1458467. doi: 10.3389/fpls.2025.1458467 (PMC11814167; doi:10.3389/fpls.2025.1458467)
Supplement: Supplementary file 1 [file Table1.docx]

# Supplementary Tables

## Supplementary Tables

Supplementary Table 1 Primers used in this study

| Primers for generating DNA vectors | |
| --- | --- |
| Primer | Sequences |
| Primers for generating DNA vectors | |
| *OsDUF868.12*-OE-F | caggtcgactctagaggatccATGCCCGAGGGGGGGATA |
| *OsDUF868.12*-OE-R | aattcgagctggtcagagctcCTAGCATTTCCAGGCGTAGACG |
| *OsDUF868.12*-GFP-F | tggagaggacagcccaagcttATGCCTGCCATGGCCAAG |
| *OsDUF868.12*-GFP-R | gtaccgaattcccggggatccTCAAGCTGCAGGTGCCTTG |
| *OsDUF868.12*-GUS-F | acctgcaggcatgcaagcttAGCAACAACAACTGCCCTCT |
| *OsDUF868.12*-GUS-R | gtaccgaattcccggggatccTCAAGCTGCAGGTGCCTTG |
| Primers for detection of knockout lines | |
| *OsDUF868.12*-detect-F | GATTACGGGTGCTACGACGA |
| *OsDUF868.12*-detect-R | GTCCTTGACCTCGGCAGAGT |
| Primers for RT-qPCR | |
| *OsSOS3*-F | CAGACAGGGTGTTTGATTTGTT |
| *OsSOS3*-R | TCATAGAGCTTGAATGCAAACG |
| *OsSOS1*-F | TTGAGATAGGGAGGCCCGAA- |
| *OsSOS1*-R | GTGCTCCTTGCTTTGTGTCC |
| *OsNHX1*-F | CGACCCACACTGTCCACTAC |
| *OsNXH1*-R | CTCTGCTCGGTTGGTGATCC |
| *OsLEA3*-F | TGAAGAGCACGGTGGTCGG |
| *OsLEA3*-R | GGCAGAGGTGTCCTTGTTGG |
| *OsRbohB*-F | TGACGAGCGTTTACGAGGAG |
| *OsRbohB*-R | ATGTGTCCTCACCCTTGTGC |
| *OsDUF868.12*-qpcr-F | AGCAGCAACGGGGAGAACC |
| *OsDUF868.12*-qpcr-R | GCATTTCCAGGCGTAGACGAG |
| *UBQ5*-F | ACCACTTCGACCGCCACTACT |
| *UBQ5*-R | ACGCCTAAGCCTGCTGGTT |
